# Correction: Associations between Social Isolation Index and changes in grip strength, gait speed, bone mineral density (BMD), and self-reported incident fractures among older adults: Results from the Canadian Longitudinal Study on Aging (CLSA)

**DOI:** 10.1371/journal.pone.0298923

**Published:** 2024-02-12

**Authors:** Ahreum Lee, Caitlin McArthur, George Ioannidis, Alexandra Mayhew, Jonathan D. Adachi, Lauren E. Griffith, Lehana Thabane, Alexandra Papaioannou

There are errors in the Funding section. The correct Funding statement is: This research was made possible using the data/biospecimens collected by the Canadian Longitudinal Study on Aging (CLSA). Funding for the Canadian Longitudinal Study on Aging (CLSA) is provided by the Government of Canada through the Canadian Institutes of Health Research (CIHR) under grant reference: LSA 94473 and the Canada Foundation for Innovation, as well as the following provinces, Newfoundland, Nova Scotia, Quebec, Ontario, Manitoba, Alberta, and British Columbia. The study has been conducted using the CLSA dataset, Baseline Comprehensive Dataset Version 4.1 and Follow-up 1 Comprehensive Dataset Version 3.0, updated Nov. 29 2021, with the CLSA Sample Weights Version 1.2., under Application Number 1906027. The CLSA is led by Drs. Parminder Raina, Christiana Wolfson and Susan Kirkland.
