# Supplementary material for: Associations between Social Isolation Index and changes in grip strength, gait speed, bone mineral density (BMD), and self-reported incident fractures among older adults: Results from the Canadian Longitudinal Study on Aging (CLSA)
Source: PLoS One. 2023 Oct 18;18(10):e0292788. doi: 10.1371/journal.pone.0292788 (PMC10584182; doi:10.1371/journal.pone.0292788)
Supplement: S1 Table — (DOCX) [file pone.0292788.s002.docx]

**S1 Table. Univariate analyses of the CLSA-SII in all participants (n=11,344)**

| **Baseline variables (covariates)** | **Social isolation index (CLSA-SII, range 0-10), mean (SD) or r** | **P-value** |
| --- | --- | --- |
| Age group (year)  65-74  75+  Sex  Males  Females | 3.32 (1.36)  3.70 (1.42)  3.26 (1.36)  3.69 (1.40) | <.001  <.001 |
| BMI (kg/m^2^) | -0.021 | 0.046 |
| Education  Less than secondary  Secondary, no post-secondary  Some post-secondary  Post-secondary | 3.77 (1.48)  3.52 (1.41)  3.64 (1.44)  3.41 (1.38) | <.001 |
| Total household income  Less than $20,000  $20,000-$49,999  $50,000-$99,999  $100,000-$149,999  $150,000+ | 4.72 (1.32)  3.86 (1.47)  3.22 (1.27)  2.96 (1.18)  2.96 (1.08) | <.001 |
| Smoking status  Current smoker  Non-smoker  Former smoker | 3.94 (1.48)  3.43 (1.38)  3.46 (1.39) | <.001 |
| Alcohol consumption  Almost every day  2-5 times a week  1-4 times a month  Less than once a month  Never | 3.36 (1.35)  3.29 (1.32)  3.51 (1.41)  3.76 (1.44)  3.70 (1.49) | <.001 |
| Self-reported osteoporosis  Yes  No | 3.73 (1.45)  3.42 (1.38) | <.001 |
| Self-reported rheumatoid arthritis  Yes  No | 3.63 (1.41)  3.46 (1.39) | 0.016 |
| Self-reported history of fractures since adulthood  Yes  No | 3.60 (1.41)  3.44 (1.39) | <.001 |
| Maternal fracture history  Yes  No | 3.46 (1.39)  3.46 (1.39) | 0.856 |
| Corticosteroid use  Yes  No | 3.50 (1.42)  3.46 (1.39) | 0.307 |
| Self-reported prior falls  Yes  No | 3.58 (1.42)  3.46 (1.40) | 0.068 |
| Diabetes  Yes  No | 3.46 (1.41)  3.47 (1.40) | 0.792 |
| The five-item Diener Satisfaction with Life Scale (SWLS, range 5-35) | -0.236 | <.001 |
| Center for Epidemiology Studies Depression 9 Scale (CES-D 9, range 0-27) | 0.204 | <.001 |
| Psychological distress (range 10-43) | 0.155 | <.001 |
| Nutritional risk (AB SCREEN II, range 0-48) | -0.266 | <.001 |
| Perceived mental health  Good/ Very good/ Excellent  Poor/ Fair | 3.44 (1.38)  4.13 (1.63) | <.001 |
| Perceived health  Good/ Very good/ Excellent  Poor/ Fair | 3.45 (1.39)  3.74 (1.51) | <.001 |
| Physical activity scale for the elderly score (PASE, range 0-629) | -0.151 | <.001 |
| Grip strength (kg) | -0.202 | <.001 |
| Gait speed (m/s) | -0.133 | <.001 |
| DXA femoral neck BMD T-score (SD) | -0.123 | <.001 |

Abbreviations: CLSA-SII=Canadian Longitudinal Study on Aging – Social Isolation Index; SD=Standard Deviation; r=Coefficient of correlation; BMI=Body Mass Index; SWLS=Satisfaction with Life Scale; CES-D 9=Center for Epidemiology Studies Depression 9 Scale; AB SCREEN II=Abbreviated Seniors in the Community Risk Evaluation for Eating and Nutrition II; PASE=Physical Activity Scale for the Elderly score; DXA=Dual-Energy X-ray absorptiometry; BMD=Bone Mineral Density

Binary and dichotomous variables were tested using a T-test, category variables were tested using Welch’s ANOVA, and continuous variables were tested using Pearson correlation.

Non-weighted results.
