# Supplementary material for: Associations between Social Isolation Index and changes in grip strength, gait speed, bone mineral density (BMD), and self-reported incident fractures among older adults: Results from the Canadian Longitudinal Study on Aging (CLSA)
Source: PLoS One. 2023 Oct 18;18(10):e0292788. doi: 10.1371/journal.pone.0292788 (PMC10584182; doi:10.1371/journal.pone.0292788)
Supplement: S3 Table — (DOCX) [file pone.0292788.s004.docx]

**S3 Table. Two-way interaction terms between CLSA-SII and age, sex, CES-D 9, SWLS and PASE in the three-year changes of grip strength, gait speed, BMD, osteoporosis classification by DXA and self-reported incident fractures in all participants**

| **Two-way interaction terms** | **β or OR (95% CI), p-value** |
| --- | --- |
| Absolute change in grip strength  CLSA-SII*Age  CLSA-SII*Sex (Ref: Males)  CLSA-SII*CES-D 9  CLSA-SII*SWLS  CLSA-SII*PASE | -0.006 (-0.020, 0.009), 0.451  0.072 (-0.114, 0.259), 0.447  0.001 (-0.021, 0.024), 0.907  0.002 (-0.013, 0.017), 0.791  -0.001 (-0.002, 0.001), 0.457 |
| Percentage change in grip strength  CLSA-SII*Age  CLSA-SII*Sex (Ref: Males)  CLSA-SII*CES-D 9  CLSA-SII*SWLS  CLSA-SII*PASE | -0.011 (-0.063, 0.041), 0.670  0.438 (-0.205, 1.081), 0.182  -0.007 (-0.085, 0.071), 0.863  -0.005 (-0.069, 0.059), 0.872  -0.001 (-0.007, 0.004), 0.608 |
| Absolute change in gait speed  CLSA-SII*Age  CLSA-SII*Sex (Ref: Males)  CLSA-SII*CES-D 9  CLSA-SII*SWLS  CLSA-SII*PASE | -0.000 (-0.001, 0.001), 0.915  0.002 (-0.005, 0.009), 0.572  0.000 (-0.001, 0.001), 0.731  -0.000 (-0.001, 0.000), 0.607  -0.000 (-0.000, -0.000), 0.047 |
| Percentage change in gait speed  CLSA-SII*Age  CLSA-SII*Sex (Ref: Males)  CLSA-SII*CES-D 9  CLSA-SII*SWLS  CLSA-SII*PASE | -0.015 (-0.078, 0.048), 0.641  0.012 (-0.726, 0.749), 0.975  0.019 (-0.067, 0.104), 0.667  -0.020 (-0.083, 0.043), 0.536  -0.005 (-0.011, 0.001), 0.127 |
| Annualized absolute (g/cm^2^) change in femoral neck BMD  CLSA-SII*Age  CLSA-SII*Sex (Ref: Males)  CLSA-SII*CES-D 9  CLSA-SII*SWLS  CLSA-SII*PASE | -0.000 (-0.000, 0.000), 0.151  0.000 (0.000, 0.001), 0.044  0.000 (-0.000, 0.000), 0.661  -0.000 (-0.000, 0.000), 0.412  0.000 (-0.000, 0.000), 0.255 |
| Annualized percentage change in femoral neck BMD  CLSA-SII*Age  CLSA-SII*Sex (Ref: Males)  CLSA-SII*CES-D 9  CLSA-SII*SWLS  CLSA-SII*PASE | -0.004 (-0.008, 0.000), 0.075  0.050 (-0.001, 0.102), 0.056  0.002 (-0.004, 0.008) 0.557  -0.002 (-0.006, 0.002), 0.408  0.000 (-0.000, 0.001), 0.299 |
| Annualized absolute (g/cm^2^) change in total hip BMD  CLSA-SII*Age  CLSA-SII*Sex (Ref: Males)  CLSA-SII*CES-D 9  CLSA-SII*SWLS  CLSA-SII*PASE | -0.000 (-0.000, 0.000), 0.613  0.000 (-0.000, 0.001), 0.228  -0.000 (-0.000, 0.000), 0.386  -0.000 (-0.000, 0.000), 0.255  -0.000 (-0.000, 0.000), 0.833 |
| Annualized percentage change in total hip BMD  CLSA-SII*Age  CLSA-SII*Sex (Ref: Males)  CLSA-SII*CES-D 9  CLSA-SII*SWLS  CLSA-SII*PASE | -0.002 (-0.006, 0.002), 0.307  0.020 (-0.018, 0.059), 0.298  -0.002 (-0.007, 0.003), 0.403  -0.002 (-0.005, 0.001), 0.255  -0.000 (-0.000, 0.000), 0.827 |
| Change for Osteoporosis classification by DXA  CLSA-SII*Age group (Ref: aged 65-74yr)  CLSA-SII*Sex (Ref: Males)  CLSA-SII*CES-D 9  CLSA-SII*SWLS  CLSA-SII*PASE | 1.05 (0.92, 1.18), 0.767  0.96 (0.84, 1.10), 0.241  0.99 (0.98, 1.00), 0.583  1.01 (0.99, 1.02), 0.641  1.00 (1.00, 1.00), 0.302 |
| Self-reported incident fractures  CLSA-SII*Age group (Ref: aged 65-74yr)  CLSA-SII*Sex (Ref: Males)  CLSA-SII*CES-D 9  CLSA-SII*SWLS  CLSA-SII*PASE | 1.10 (0.89, 1.37), 0.383  0.95 (0.74, 1.24), 0.726  1.02 (1.00, 1.04), 0.013  0.99 (0.97, 1.01), 0.324  1.00 (1.00, 1.00), 0.429 |

CLSA-SII=Canadian Longitudinal Study on Aging – Social Isolation Index; CES-D 9=Center for Epidemiology Studies Depression 9 Scale; SWLS=Satisfaction with Life Scale; PASE=Physical Activity Scale for the Elderly score; BMD=Bone Mineral Density; DXA=Dual-Energy X-ray absorptiometry; 95% CI=95% Confidence Interval; OR=Odds Ratio

Two-way interaction terms were tested in the Model 3, which adjusted for all covariates (i.e., age, sex, education, body mass index (BMI), total household income, smoking status, alcohol consumption, self-reported osteoporosis, self-reported rheumatoid arthritis, self-reported history of fractures since adulthood, maternal fracture history, corticosteroid use, self-reported prior falls, diabetes, DXA femoral neck BMD T-score, grip strength, gait speed, the five-item diener satisfaction with life scale (SWLS), centre for epidemiological studies depression scale (CES-D 9), psychological distress, nutritional risk (AB SCREEN II), perceived mental health, perceived health, and physical activity scale for the elderly (PASE))

Weighted results, v1.2.
