# Supplementary material for: Associations between Social Isolation Index and changes in grip strength, gait speed, bone mineral density (BMD), and self-reported incident fractures among older adults: Results from the Canadian Longitudinal Study on Aging (CLSA)
Source: PLoS One. 2023 Oct 18;18(10):e0292788. doi: 10.1371/journal.pone.0292788 (PMC10584182; doi:10.1371/journal.pone.0292788)
Supplement: S4 Table — (DOCX) [file pone.0292788.s005.docx]

**S4 Table. Results of multivariable linear, logistic, multinomial logistic regression models of CLSA-SII as risk factors of the three-year changes in grip strength, gait speed, BMD, osteoporosis by DXA and self-reported incident fractures in all participants**

|  | **CLSA-SII (per unit one change)** | |
| --- | --- | --- |
| **Three-year change** | **Model 1**  **Unadjusted β or OR (95% CI), p-value** | **Model 2**  **Fully adjusted* β or OR (95% CI), p-value** |
| Grip strength (kg)  Absolute change  Percentage change | 0.039 (-0.033, 0.112), 0.289  -0.096 (-0.359, 0.166), 0.469 | 0.077 (-0.019, 0.174), 0.117  0.254 (-0.093, 0.601), 0.151 |
| Gait speed (m/s)  Absolute change  Percentage change | -0.0004 (-0.003, 0.002), 0.774  -0.086 (-0.388, 0.215), 0.574 | 0.0007 (-0.003, 0.004), 0.711  -0.435 (-0.271, 0.400), 0.307 |
| Annualized absolute (g/cm^2^) change in femoral neck BMD | -0.0001 (-0.000, 0.000), 0.127 | -0.0000 (-0.000, 0.000), 0.682 |
| Annualized percentage change in femoral neck BMD | -0.023 (-0.046, -0.001), 0.045 | -0.004 (-0.034, 0.027), 0.807 |
| Annualized absolute (g/cm^2^) change in total hip BMD | -0.0002 (-0.000, -0.000), 0.021 | 0.0000 (-0.000, 0.000), 0.576 |
| Annualized percentage change in total hip BMD | -0.028 (-0.045, -0.010), 0.002 | 0.008 (-0.014, 0.031), 0.463 |
| Change for osteoporosis classification by DXA†  Unchanged  Worsened  Improved | Ref  1.08 (1.02, 1.14), 0.006  1.06 (0.96, 1.16), 0.251 | Ref  1.02 (0.95, 1.10), 0.561  1.00 (0.89, 1.13), 0.980 |
| Self-reported incident fractures‡  Worsened  Improved/Same | 1.18 (1.09, 1.27), <.001  Ref | 1.13 (1.00, 1.27), 0.053  Ref |

CLSA-SII=Canadian Longitudinal Study on Aging – Social Isolation Index; SE=Standard Error; BMD=Bone Mineral Density; DXA=Dual-Energy X-ray absorptiometry

*Fully adjusted for age, sex, education, body mass index (BMI), total household income, smoking status, alcohol consumption, self-reported osteoporosis, self-reported rheumatoid arthritis, self-reported history of fractures since adulthood, maternal fracture history, corticosteroid use, self-reported prior falls, diabetes, DXA femoral neck BMD T-score, grip strength, gait speed, the five-item diener satisfaction with life scale (SWLS), centre for epidemiological studies depression scale (CES-D 9), psychological distress, nutritional risk (AB SCREEN II), perceived mental health, perceived health, and physical activity scale for the elderly (PASE)

Model 1 was unadjusted and Model 2 was fully adjusted.

†Change for osteoporosis classification by DXA from baseline to follow-up were classified as follows: 1) unchanged (normal to normal, osteopenia to osteopenia, or osteoporosis to osteoporosis), 2) worsened (normal to osteopenia, normal to osteoporosis, or osteopenia to osteoporosis), and 3) improved (osteopenia to normal, osteoporosis to normal, or osteoporosis to osteopenia)

‡Self-reported incident fractures from baseline to three-year follow-up were classified as follows: 1) worsened (no prior fractures to incident fractures, or prior fractures to incident fractures) and 2) improved/same (prior fractures to no incident fractures, or no prior fractures to no incident fractures)

Non-weighted results.
