# Supplementary material for: Associations between Social Isolation Index and changes in grip strength, gait speed, bone mineral density (BMD), and self-reported incident fractures among older adults: Results from the Canadian Longitudinal Study on Aging (CLSA)
Source: PLoS One. 2023 Oct 18;18(10):e0292788. doi: 10.1371/journal.pone.0292788 (PMC10584182; doi:10.1371/journal.pone.0292788)
Supplement: S5 Table — (DOCX) [file pone.0292788.s006.docx]

**S5 Table. Two-way interaction terms between CLSA-SII and age, sex, CES-D, SWLS and PASE in the three-year changes of grip strength, gait speed, BMD, osteoporosis classification by DXA and self-reported incident fractures**

| **Two-way interaction terms** | **β or OR (95% CI), p-value** |
| --- | --- |
| Absolute change in grip strength  CLSA-SII*Age  CLSA-SII*Sex (Ref: Males)  CLSA-SII*CES-D 9  CLSA-SII*SWLS  CLSA-SII*PASE | 0.006 (-0.010, 0.021), 0.463  0.035 (-0.142, 0.211), 0.699  -0.003 (-0.024, 0.018), 0.773  0.019 (0.005, 0.034), 0.010  -0.000 (-0.002, 0.001), 0.782 |
| Percentage change in grip strength  CLSA-SII*Age  CLSA-SII*Sex (Ref: Males)  CLSA-SII*CES-D 9  CLSA-SII*SWLS  CLSA-SII*PASE | 0.019 (-0.036, 0.075), 0.494  0.125 (-0.509, 0.759), 0.670  -0.016 (-0.093, 0.060), 0.674  0.067 (0.015, 0.120), 0.012  -0.001 (-0.006, 0.005), 0.801 |
| Absolute change in gait speed  CLSA-SII*Age  CLSA-SII*Sex (Ref: Males)  CLSA-SII*CES-D 9  CLSA-SII*SWLS  CLSA-SII*PASE | -0.000 (-0.001, 0.000), 0.231  -0.005 (-0.012, 0.001), 0.110  0.000 (-0.000, 0.001), 0.354  -0.000 (-0.001, 0.000), 0.660  0.000 (-0.000, 0.000), 0.283 |
| Percentage change in gait speed  CLSA-SII*Age  CLSA-SII*Sex (Ref: Males)  CLSA-SII*CES-D 9  CLSA-SII*SWLS  CLSA-SII*PASE | -0.057 (-0.120 to 0.006), 0.075  -0.558 (-1.274, 0.157), 0.126  0.034 (-0.052, 0.121), 0.439  0.002 (-0.058, 0.062), 0.948  0.004 (-0.002, 0.011), 0.176 |
| Annualized absolute (g/cm^2^) change in femoral neck BMD  CLSA-SII*Age  CLSA-SII*Sex (Ref: Males)  CLSA-SII*CES-D 9  CLSA-SII*SWLS  CLSA-SII*PASE | -0.000 (-0.000, 0.000), 0.847  0.000 (0.000, 0.001), 0.007  0.000 (-0.000, 0.000), 0.052  -0.000 (-0.000, 0.000), 0.071  -0.000 (-0.000, 0.000), 0.967 |
| Annualized percentage change in femoral neck BMD  CLSA-SII*Age  CLSA-SII*Sex (Ref: Males)  CLSA-SII*CES-D 9  CLSA-SII*SWLS  CLSA-SII*PASE | -0.001 (-0.006, 0.004), 0.641  0.070 (0.014, 0.126), 0.014  0.008 (0.001, 0.014), 0.029  -0.005 (-0.010, -0.000), 0.038  0.000 (-0.000, 0.000), 0.987 |
| Annualized absolute (g/cm^2^) change in total hip BMD  CLSA-SII*Age  CLSA-SII*Sex (Ref: Males)  CLSA-SII*CES-D 9  CLSA-SII*SWLS  CLSA-SII*PASE | 0.000 (-0.000, 0.000), 0.373  0.000 (0.000, 0.001), 0.045  -0.000 (-0.000, 0.000), 0.887  -0.000 (-0.000, 0.000), 0.060  0.000 (-0.000, 0.000), 0.669 |
| Annualized percentage change in total hip BMD  CLSA-SII*Age  CLSA-SII*Sex (Ref: Males)  CLSA-SII*CES-D 9  CLSA-SII*SWLS  CLSA-SII*PASE | 0.001 (-0.003, 0.004), 0.649  0.035 (-0.006, 0.076), 0.091  0.000 (-0.005, 0.005), 0.954  -0.004 (-0.007, -0.000), 0.031  0.000 (-0.000, 0.000), 0.583 |
| Change for osteoporosis classification by DXA  CLSA-SII*Age group (Ref: aged 65-74yr)  CLSA-SII*Sex (Ref: Males)  CLSA-SII*CES-D 9  CLSA-SII*SWLS  CLSA-SII*PASE | 1.05 (0.92, 1.21), 0.142  0.96 (0.83, 1.10), 0.091  0.99 (0.98, 1.01), 0.068  1.00 (0.99, 1.01), 0.698  1.00 (1.00, 1.27), 0.712 |
| Self-reported incident fractures  CLSA-SII*Age group (Ref: aged 65-74yr)  CLSA-SII*Sex (Ref: Males)  CLSA-SII*CES-D 9  CLSA-SII*SWLS  CLSA-SII*PASE | 1.16 (0.93, 1.45), 0.180  1.12 (0.88, 1.42), 0.352  1.02 (1.00, 1.05), 0.063  0.99 (0.98, 1.01), 0.599  1.00 (1.00, 1.00), 0.223 |

CLSA-SII=Canadian Longitudinal Study on Aging – Social Isolation Index; CES-D 9=Center for Epidemiology Studies Depression 9 Scale; SWLS=Satisfaction with Life Scale; PASE=Physical Activity Scale for the Elderly score; BMD=Bone Mineral Density; DXA=Dual-Energy X-ray absorptiometry; 95% CI=95% Confidence Interval; OR=Odds Ratio

Two-way interaction terms were tested in the Model 3, which adjusted for all covariates (i.e., age, sex, education, body mass index (BMI), total household income, smoking status, alcohol consumption, self-reported osteoporosis, self-reported rheumatoid arthritis, self-reported history of fractures since adulthood, maternal fracture history, corticosteroid use, self-reported prior falls, diabetes, DXA femoral neck BMD T-score, grip strength, gait speed, the five-item diener satisfaction with life scale (SWLS), centre for epidemiological studies depression scale (CES-D 9), psychological distress, nutritional risk (AB SCREEN II), perceived mental health, perceived health, and physical activity scale for the elderly (PASE))

Non-weighted results.
