# Supplementary material for: Associations between Social Isolation Index and changes in grip strength, gait speed, bone mineral density (BMD), and self-reported incident fractures among older adults: Results from the Canadian Longitudinal Study on Aging (CLSA)
Source: PLoS One. 2023 Oct 18;18(10):e0292788. doi: 10.1371/journal.pone.0292788 (PMC10584182; doi:10.1371/journal.pone.0292788)
Supplement: Attachment — Submitted filename: Response to reviewers (ver.1).docx [file pone.0292788.s007.docx]

To the Editor:

We appreciate the opportunity to resubmit the manuscript as a new submission following the concerns raised during the initial review process.

First and foremost, we would like to acknowledge and apologize for an oversight in our previous submission. Regrettably, we inadvertently omitted the initial approval letter from the local ethics committee, as well as subsequent annual review approvals. This oversight has now been rectified, and we have included the appropriate documentation in this revised submission.

Below, please finds a point-by-point response to the editor’s comment, which are included verbatim. A page and a paragraph number refer to location in the revised manuscript.

**AN EDITOR COMMENT**

A COMMENT:

Based on our evaluation of your manuscript and the information you provided, we do not feel that your submission meets our ethical requirements for human subjects research submissions. PLOS ONE requires that research meets all applicable standards for the ethics of experimentation and research integrity (<http://journals.plos.org/plosone/s/human-subjects-research>). We reserve the right to reject any submission that does not meet our internal ethical standards, which in some cases are more stringent than local ethical standards.

We recognize that you obtained ethical approval from your local ethics committee after you accessed the data for the study: your ethics approval document is valid from October 17 2021 to October 16 2022, while you have accessed the data on October 13th 2019. For human subjects research, PLOS ONE requires that authors obtain ethics committee approval before beginning the study or accessing the data, and we do not accept retrospective ethical approval.

A RESPONSE:

We appreciate the significant point for ethical approval and data access. We would like to clarify the approval process for our research was conducted in accordance with the regulations set by the local ethical approval on December 6^th^, 2019, and subsequent annual review approvals were obtained as per the committee’s requirements. We have attached the relevant approval letters to this resubmission, providing a comprehensive overview of the approval history and the dates of data access.

We have attached the missing approval letters for your reference:

- Date of data access (start date of study): Marche 25th, 2020
- Final ethics approval: December 6th, 2019 – December 6th, 2020
- Annual review approval (1st): December 6th, 2020 – December 6th, 2021
- Annual review approval (2nd): December 6th, 2021 – December 6th, 2022
- Annual review approval (3rd): December 6th, 2022 – December 6th, 2023

To address this point, we have revised the following to the method section:

*“This study received research ethics board approval from Hamilton Integrated Research Ethics Board on December 6th, 2019, and the participants provided their written informed consent to participate in the CLSA. We used a de-identification record in order to prevent identification of individuals which was accessed on March 25th, 2020.”* (Page 10, Para 5, Line 224-229)
